# Substrate Dependent Ad-Atom Migration on Graphene and the Impact on Electron-Beam Sculpting Functional Nanopores

**DOI:** 10.3390/s17051091

**Published:** 2017-05-10

**Authors:** Kevin J. Freedman, Gaurav Goyal, Chi Won Ahn, Min Jun Kim

**Affiliations:** 1 Joint Genome Institute, Lawrence Berkeley National Laboratory, Walnut Creek, CA 94598, USA; kjf322@gmail.com; 2 Global Viral, 425 California St., San Francisco, CA 90104, USA; 3 Quantum Biosystems, 1455 Adams Dr., Menlo Park, CA 94025, USA; virgaurav@gmail.com; 4 Nano-Materials Laboratory, National Nanofab Center, Daejeon 305-806, Korea; cwahn@nnfc.re.kr; 5 Department of Mechanical Engineering, Southern Methodist University, Dallas, TX 75275, USA

**Keywords:** nanopore, graphene, DNA, sequencing, single molecule detection, DNA detection, ad-atom

## Abstract

The use of atomically thin graphene for molecular sensing has attracted tremendous attention over the years and, in some instances, could displace the use of classical thin films. For nanopore sensing, graphene must be suspended over an aperture so that a single pore can be formed in the free-standing region. Nanopores are typically drilled using an electron beam (e-beam) which is tightly focused until a desired pore size is obtained. E-beam sculpting of graphene however is not just dependent on the ability to displace atoms but also the ability to hinder the migration of ad-atoms on the surface of graphene. Using relatively lower e-beam fluxes from a thermionic electron source, the C-atom knockout rate seems to be comparable to the rate of carbon ad-atom attraction and accumulation at the e-beam/graphene interface (i.e., R_knockout_ ≈ R_accumulation_). Working at this unique regime has allowed the study of carbon ad-atom migration as well as the influence of various substrate materials on e-beam sculpting of graphene. We also show that this information was pivotal to fabricating functional graphene nanopores for studying DNA with increased spatial resolution which is attributed to atomically thin membranes.

## 1. Introduction

The guided migration of biological molecules through a pore and their real-time electrical detection is the fundamental premise of most nanopore-based sensors [1,2,3,4,5]. Driven by the anticipation of developing the next high-throughput, long-read DNA sequencing technology, the materials and fabrication techniques for making nanopores has also grown quite dramatically [6,7,8]. The basic principle of nanopore sensing is that a molecular species will block a certain amount of ions while inside a pore based on physiochemical properties including but not limited to the molecule’s hydrodynamic volume, charge, shape, stability, and orientation [9,10,11,12,13]. Ideally, molecules will partition from bulk solution, reside in the pore, and exit again within a short amount of time (hundreds of microseconds) so that multiple translocation events can be recorded and statistical analysis can be performed on the electrical signatures originating from the same molecular species [14,15]. The nanopore not only acts to limit the flow of ions to a singular region on a membrane but also plays an important role in how fast a molecule traverses the pore due to pore-molecule interactions [16,17]. Aside from DNA sequencing, nanopores have also seen utility in nucleic acid analysis [18,19], plasmonic-based sensing [20,21], lipid bilayer membrane biophysical measurements [22], and protein sensing [12,23,24,25], among others.

The geometry of the nanopore is the most fundamental aspect of a nanopore experiment and should be tailored to the molecule being sensed. In most cases, changes in the material, or more commonly, the fabrication technique, will result in a change in pore geometry [26,27,28,29]. The most common free-standing solid-state membranes used for nanopore fabrication is silicon-based materials such as silicon nitride or silicon dioxide [9,30,31]. Methods of creating individual nanopores in glass capillaries [32] and track-etched polymers also exist [33], yet are not as widespread as the use of silicon-based platforms which can be fabricated using manufacturing principles from the microelectronics industry. With limitations on how thin silicon nitride could be deposited and maintain mechanical stability, local thinning of silicon nitride was devised and shown to be quite successful at achieving superior resolution [34]. An alternative to thinning a local region of a silicon-based membrane is to use graphene which is a 2D material with advantageous mechanical, electrical, and thermal properties [35]. In 2010, three groups simultaneously published their data on graphene-based DNA sensing [36,37,38] and since then many others have also demonstrated graphene’s molecular sensing capabilities [27,39,40]. Most exciting of all, graphene, and more broadly 2D materials, are capable of probing molecules in a unique way that was not possible using silicon-based materials.

The process of nanopore drilling can be undertaken using a focused ion beam [41] for pore sizes >20 nm, or a field-emission transmission electron microscope (TEM) [28] for pore sizes in the range of 1–30 nm. Focused electron beams are capable of direct atomic displacement via “knock-on” collisions [42]. Direct atom displacement occurs when an electron collides with the nucleus of an atom provided that the energy transferred to the atom exceeds the local binding energy. Due to the enormous mass difference, direct collisions rarely occur in practice. In order to make sculpting more effective, electron accelerating voltages much higher than the knock-out threshold are used in combination with field-emission electron sources which have high beam fluxes (i.e., intensity). In comparison, thermionic electron sources such as lanthium hexaboride (LaB_6_) have much lower electron beam fluxes that are typically not intense enough to drill nanopores in a reasonable amount of time [27]. To our knowledge the only publication to show nanopore drilling capabilities using a thermionic source is from our group using single and multi-layer graphene as the sculpting material [27,43]. Not only was drilling possible in graphene using a thermionic source, but nanopores could be shrunk using electron beam induced deposition (EBID) of carbon. We further showed that the amorphous carbon deposited through the EBID process could be transformed into graphitic structures at the edge of the pore [27].

In the current work, graphene nanopore drilling kinetics are investigated using various support structures to determine if electron beam induced heating plays a role in nanopore formation. Previous reports of graphene nanopore fabrication have been demonstrated with silicon nitride membranes using a single FIB-drilled aperture (200 nm) on which the graphene was suspended [38]. Surprisingly, the same support structure was impossible to drill in using lower current densities (using a LaB_6_ electron source). Based on the knock-on dislocation model of graphene drilling, this result was anomalous since momentum transfer to a local region should be independent of surrounding material and should only extend the time needed to form a nanopore. Despite focusing the electron beam to a small nanometer-scale point on the graphene membrane, the structure and composition of the support material hundreds of nanometers away are found to still influence the success rate of nanopore fabrication. Carbon and silicon dioxide coatings were applied to silicon nitride to investigate the phenomenon in more detail. Owing to the renewed interest in graphene/electron-beam interactions in recent years due to graphene-based DNA sequencing, substrate-dependent drilling in graphene will guide further work in the field of nanopore-based biosensing.

## 2. Materials and Methods 

### 2.1. Fabrication of Silicon Nitride Supporting Structure

Apertures were drilled in a 50 nm thick free-standing silicon nitride membrane which was supported on all sides by a silicon chip (5.5 × 5.5 mm^2^). Fabrication of this membrane consisted of first depositing a layer of low-stress silicon nitride on a silicon wafer using low pressure chemical vapor deposition (LPCVD) followed by photolithography, deep reactive ion etching (DRIE) and KOH etching to form a 50 × 50 μm^2^ square membrane. Silicon nitride apertures were then drilled using a focused ion beam (FIB, Strata DB235, FEI, Hillsboro, OR, USA). Different aperture sizes were made by modifying the ion beam drill time. Once the graphene was transferred over the aperture, all graphene drilling experiments used a JEOL JEM 2100 instrument (JEOL Ltd., Tokyo, Japan) equipped with a LaB_6_ electron source. Although both aperture size and graphene pore size could be imaged at the same time, some apertures were also characterized according to their resistance prior to graphene transfer. Using 0.1 M KCl, the resistance measurements of 205, 419, 598, 800 nm SiN apertures are 4907.2, 2101.6, 1476.1, 1003.9 kOhms, respectively.

### 2.2. Graphene Transfer to Substrate

We used a graphene transfer process similar to those reported by Garaj et al. [38] Briefly, graphene was grown by CVD on a copper substrate followed by spin coating a polymer (polymethyl-methacrylate, PMMA) on the surface [15]. The copper substrate was then etched using ferric chloride thereby releasing the graphene. A new support (silicon nitride chip or carbon grid) was then used to scoop up the floating graphene/PMMA. PMMA was then removed using thorough solvent washes including a 30 min soak in heated acetone. 

### 2.3. Substrate Carbon Coating

A 108C Auto Carbon Coater (Ted Pella Inc., Redding, CA, USA) was used to deposit carbon on a pre-drilled silicon nitride aperture of various sizes. Deposition time was optimized to obtain a 20 nm coating on the surface of the silicon nitride. The deposition thickness was verified by transmission electron microscopy of the aperture edges. 

### 2.4. Substrate Silicon Dioxide Coating

Silicon dioxide was deposited on a pre-drilled aperture using a previously published silica coating procedure [44]. The sol–gel wet chemical method was used in order to coat all pore and membrane surfaces thereby increasing the thickness of the membrane and reducing the pore diameter. Briefly, 1μL of APTES (#A3648, Sigma-Aldrich, St. Louis, MO, USA) was added to 500 μL of DI water and the reaction tube was placed into ice for 5 min. TEOS (Sigma-Aldrich, #333859) was then added to the APTES solution to carry out the silica growth reaction. For controlling coating thickness, the concentration of TEOS was altered until the desired thickness was achieved (final concentration of 25 μM in 500 μL reaction). After being mixed, the solution was cooled on ice again over a period of 20 min. Finally, the reaction tube was allowed to sit at room temperature for 30 min without any stirring. The reaction was finally stopped by washing the nanopore chip with DI water, 100% ethanol, and then allowed to dry.

### 2.5. Ionic Current Measurements

Pore characterization and event recording were accomplished by placing the graphene nanopore between two electrolytic half cells filled with buffered potassium chloride (1M). The nanopore chip was held in place using a custom built polycarbonate flow cell with PDMS gaskets to assure that the only path of ionic current is through the nanopore. Electrodes (Ag/AgCl) were placed in both chambers and connected to the headstage of a patch clamp amplifier (Axopatch 200B, Molecular Devices Inc., Sunnyvale, CA, USA) which allowed the ionic current to be measured at various applied voltages. Signals were recorded at a sampling frequency of 250 kHz and a lowpass Bessel filter of 10 kHz. For detection experiments, 48.5 kbp λ-DNA was added to the cis chamber and a voltage was applied across the nanopore.

## 3. Results

**Figure 1:**
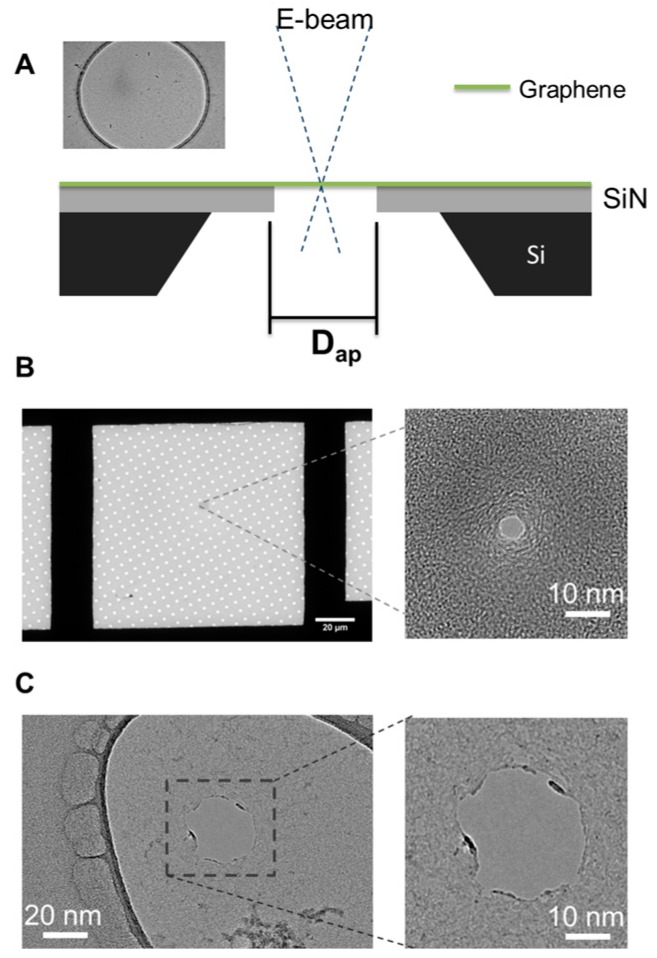
Bare substrate materials used for the suspension of graphene. (**A**) Silicon nitride (SiN) free-standing membrane (50 nm) with an aperture of various diameters. (**B**) Quantifoil^®^ holey carbon film (~10 nm) with an array of apertures (size of each aperture is 1.2 µm). Inset: a 5 nm diameter graphene pore formed using the Quantifoil^®^ apertures. (**C**) Unstructured holey carbon film (~10 nm) with a wide size distribution of apertures. Inset: 50 nm graphene nanopore formed on the holey carbon aperture.

The irradiation of free-standing graphene membranes with a focused electron beam has been used quite broadly over recent years to sculpt nanometer-scale structures. The principle behind electron beam (e-beam) sculpting, in graphene as well as silicon nitride, is direct atomic displacement. The knock-out threshold energy for C-atoms is ~80 keV [45] and so the 200 keV accelerating voltage used in this study should provide more than enough energy for atomic displacement. With the e-beam focused on a graphene membrane, it would be expected that surrounding structures would have little effect on pore formation. However during the course of optimizing e-beam conditions for nanopore sculpting, it was found that nanopore formation was highly dependent on structures hundreds of nanometers away from the focused e-beam. The substrates that were used in this study include a 50 nm silicon nitride (SiN) membrane (Figure 1A), a Quantifoil^®^ carbon film (Ted Pella Inc., Redding, CA, USA, Figure 1B), and a holey carbon grid used for TEM (Figure 1C).

**Figure 2:**
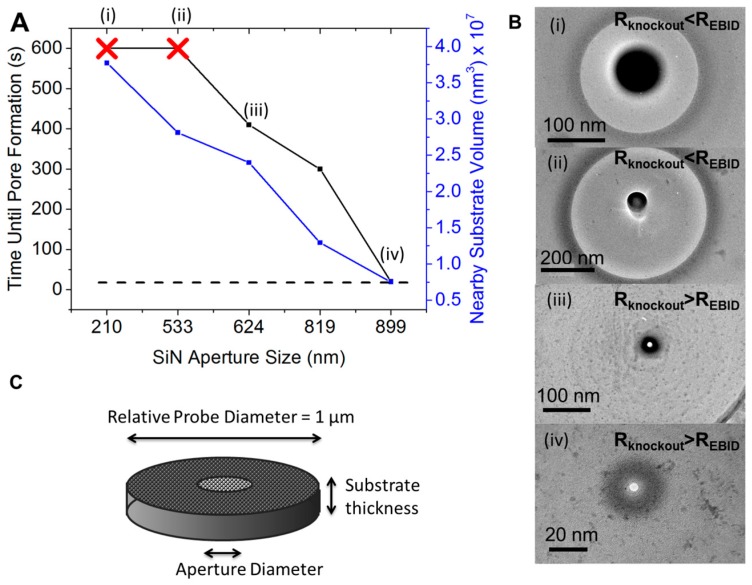
E-beam drilling times for graphene supported by SiN apertures of various sizes. (**A**) Time required for pore formation in graphene as a function of SiN aperture size. Note that smaller pores led to unsuccessful pore formation even after 10 min of focused e-beam exposure. (**B**) TEM images of the unsuccessful and successful pores that were formed in graphene. (**C**) Geometric model used to quantify the volume of support material that is in contact with the graphene. The nearby substrate material (“y-axis of (**A**)) was found by taking the volumetric difference of the probed area and the amount of material removed to form the aperture.

Prior to graphene transfer, apertures of various sizes were drilled into the SiN membranes using a focused ion beam. After graphene transfer, nanopore chips were loaded into a TEM using a custom built holder for visualization and drilling of the graphene membrane. Since the probability of having a defect or tear in the graphene increases with aperture size, it seemed advantageous to make small apertures for suspending the graphene. Despite this purported advantage, it became clear that nanopore formation was not possible at aperture sizes below ~600 nm when suspended on a SiN substrate. Instead of a nanopore forming, after 10 min, a dark circular spot was formed directly where the e-beam was focused (Figure 2A,B). As the SiN aperture was increased further, the dark circular spot became less dark and pores could eventually be formed. In order to quantify how much substrate material is in the vicinity of the graphene, a 1 µm probe diameter was arbitrarily chosen to quantify the relative amount of SiN present (Figure 2C).

When the SiN aperture was ~900 nm, the e-beam drilling kinetics and the time to form the initial pore was equivalent to that of the holey carbon films (dotted line in Figure 2A). Interestingly, pore formation did not depend at all on aperture size when the graphene was supported by the holey carbon films. In other words, SiN apertures >900 nm and all aperture sizes in the carbon films, nanopores could be formed in ~20 s. For this reason, the range of e-beam/graphene interactions, or more specifically, e-beam induced heating, may extend ~900 nm from the center of the e-beam. Several kinetic processes could be responsible for the observed effect. It was shown previously that pore formation is countered by the accumulation of amorphous carbon near the focused e-beam [27,43,46]. The source of carbon could be from knockout C-atoms or from residual hydrocarbons on the surface of the graphene. Unlike more common EBID processes, catalysis reagents are not pumped into the sample chamber and, in fact, the high vacuum inside the sample chamber ensures that no C-atoms are being deposited from the gas phase but rather originate from the graphene surface. A more accurate description of the phenomenon in this study is e-beam induced surface migration. Nevertheless, the ad-atoms on the graphene surface migrate towards the e-beam either due to electrostatic or magnetic attraction. The movement of ad-atoms on the surface depends on the 2D diffusion coefficient of C-atoms which is temperature dependent. Elevated temperatures are not required, however, and occur even at room temperature [47]. 

When the e-beam is focused on the graphene membrane, for example prior to pore formation, heating is expected and will contribute to higher ad-atom diffusion. The knockout threshold energy on the other hand is not temperature dependent and in this work we assume the knockout rate is kept constant. Therefore we believe the rate of ad-atom accumulation via the EBID process is the determining factor in whether a nanopore can be formed. For SiN aperture sizes <600 nm, the C ad-atoms re-occupy the vacancies left from the knockout process and instead an accumulation of mass is observed.

**Figure 3:**
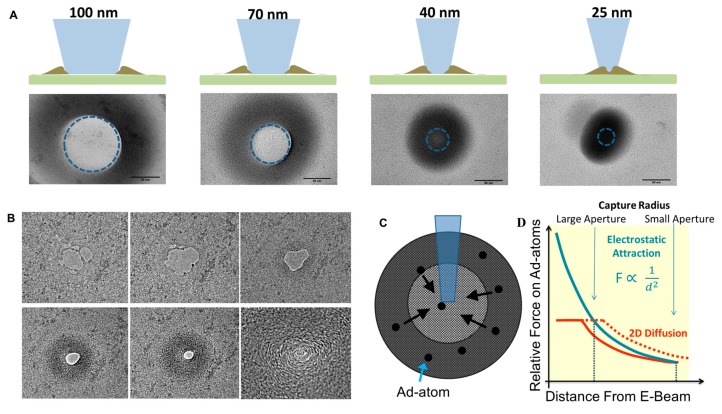
E-beam induced migration of carbon ad-atoms on the surface of graphene. (**A**) Carbon accumulation at the site of beam exposure as a function of beam size. Note that beam intensity also is reduced as the beam size becomes smaller. (**B**) E-beam induced healing of secondary holes and jagged nanopore edges by the accumulation of amorphous carbon which then can be turned into crystalline graphite under e-beam exposure. (**C**) A graphic presentation of e-beam induced migration of ad-atoms on a supported graphene sheet. (**D**) Theoretical representation of two competing forces which dictate how an ad-atom move on graphene: (1) temperature-dependent diffusion which must be overcome by (2) e-beam electrostatic attraction of ad-atoms. The point at which these two forces equal is the 2D capture radius of ad-atoms and is expected to decrease in size with larger apertures (as seen with SiN apertures).

The attraction of ad-atoms towards the e-beam was studied in more detail by altering the beam size on a given substrate. The largest of the e-beams created a ring of carbon build up with no accumulation directly in the path of the e-beam. As the beam was focused tighter, the ring of carbon turned into a single circular spot on the order of ~100 nm in diameter. Ad-atom attraction towards the e-beam is a useful phenomenon for nanofabrication as it can allow the self-repair or healing of the graphene membrane. A pore with jagged edges or even secondary holes can be filled in by the carbon atoms on the surface of the graphene [46]. Furthermore, the pore size can be fine-tuned by shrinking the diameter to the size required for biological sensing of molecules. Figure 3B shows a time series set of images of a pore with multiple secondary holes being healed by the buildup of amorphous carbon. After the amorphous carbon is used to sculpt the nanopore, the amorphous regions can be turned into crystalline material by e-beam exposure as previously shown [27]. The transition from amorphous carbon to crystalline carbon is the result of e-beam induced heating however the exact change in temperature is not known. Electron beams have been used in the past to simulate thermal diffusion and subsequently image defect diffusion in graphene [48]. 

The ability of the e-beam to capture ad-atoms will be the primary factor in whether a nanopore can be drilled. The rate of capture must be less than the knockout rate in order for a pore to be formed. The parameters that are not changing are the knockout rate and the force on the ad-atoms since the e-beam intensity is not changed. Therefore the capturing of ad-atoms occurs when the attractive force overcomes random diffusion, much like the DNA capture process in nearly all nanopore experiments. A typical nanopore experiments uses a voltage bias to electrophoretically attract DNA towards the nanopore. However, this only occurs in practice when the molecule comes close enough to the pore to overcome diffusion. Although the movement of carbon ad-atoms across graphene due to an e-beam is not fully understood, it seems that electrostatic or magnetic attraction forces play a role [49,50]. Both formulations dictate that the distance dependence of the attractive force scales according to $\frac{1}{d^{2}}$, where d is the distance between the ad-atom and the e-beam. The diffusion of ad-atoms which must be overcome is temperature dependent and therefore the propagation of heat through freestanding and supported graphene must be considered. The heat conductivity of graphene is extremely high at over 2000 W·mK^−1^ at room temperature [51]. Comparatively, silicon nitride’s thermal conductivity is on the order of <1 W·mK^−1^ [52]. Although primary heat dissipation will be conduction through graphene, heat will also be transferred at the SiN-graphene interface as well radially dispersed in the graphene. The free-standing portion of the graphene is expected to have very little temperature change due to the high conductivity and no contact with other material. It is only when the graphene is supported that temperature and therefore the diffusion barrier will be reduced. Increasing the SiN aperture thereby reduces the capture radius of the e-beam by preventing alternative routes of heat dissipation (Figure 3C).

**Figure 4:**
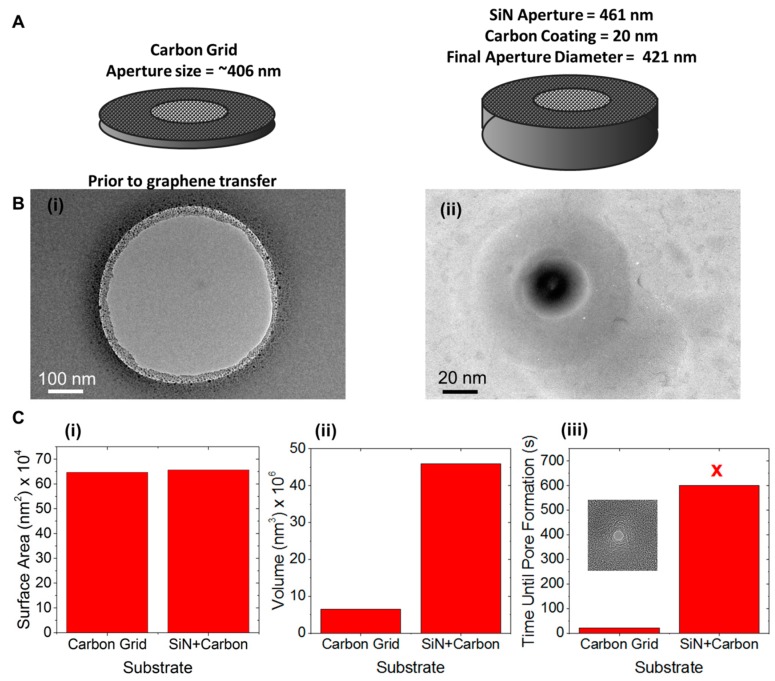
(**A**) Illustration of the two substrates used to study the influence of substrate surface area and volume on graphene e-beam sculpting kinetics. A carbon grid with aperture of ~406 nm is compared to a carbon-coated SiN membrane with an aperture of 421 nm. (**B**) TEM micrograph of the carbon-coated SiN aperture prior to graphene transfer (i) and after 10 min of failure to form a nanopore (ii). (**C**-i) Surface area that graphene is in contact with the SiN-carbon aperture within the 1 µm probe area. (**C**-ii) Volume of nearby substrate material for the holey carbon grid and the carbon-coated SiN aperture. (**C**-iii) Time to form a nanopore when graphene is transferred to a holey carbon film and a carbon-coated SiN aperture.

An alternative hypothesis is that the surface properties of the substrate (e.g., surface energy, surface charge) are affecting carbon ad-atom diffusion. To test this hypothesis, a SiN aperture was coated with carbon and the drilling kinetics were compared that of a carbon film (~10 nm in thickness; Figure 4A). The aperture size was 406 nm and 421 nm respectively so the surface area of graphene (freestanding versus supported) was similar while the amount of support material (i.e., volume) was much greater for the carbon coated SiN aperture. Pore formation in graphene supported by a thin carbon grid was already shown to occur within 20 s and with almost no accumulation of ad-atoms. However the SiN aperture coated with carbon could not successfully be used for drilling a graphene nanopore (Figure 4B). After 10 min of attempting to drill a pore, it was clear that the rate of ad-atom accumulation was greater than the knockout rate. Since the main difference in the two substrates was the amount of material that can participate in heat dissipation, the amount of volume near the graphene seems to be a key factor in graphene nanopore formation.

**Figure 5:**
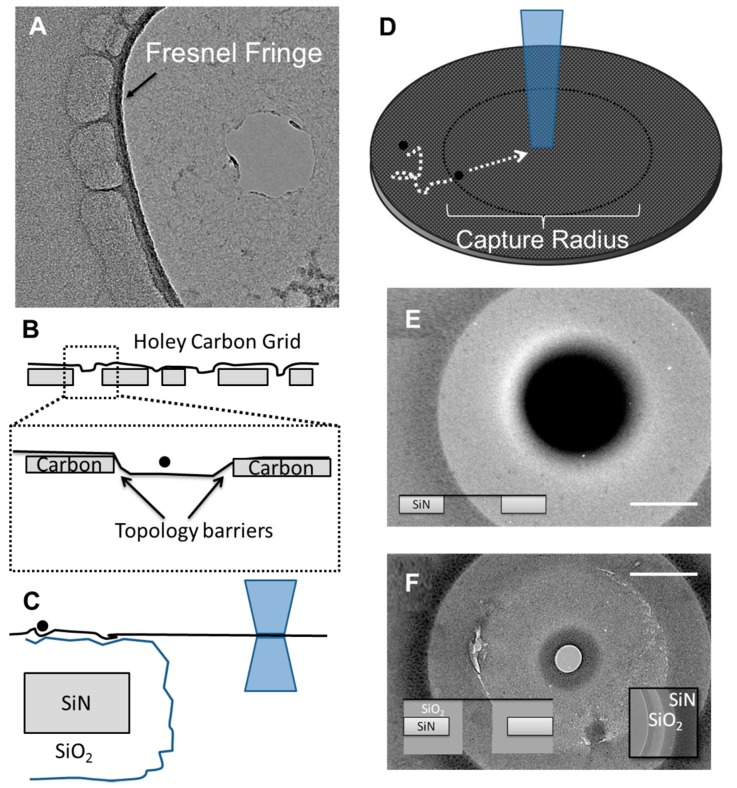
(**A**) TEM image showing that the graphene nanopore lies on a different focal plane as evidenced by the stronger Fresnel fringe next to the carbon film. (**B**) The expected surface topology of the graphene laying on top of a holey carbon film with many apertures. (**C**) Schematic of the increasing volume associated with the coating of SiN with SiO_2_ and also the surface topology which may hinder ad-atom diffusion. (**D**) Schematic of the transition from 2D diffusion of surface ad-atoms on graphene to electrostatic attraction towards the e-beam. Inset: schematic of the graphene suspended on a SiN aperture. (**E**) TEM image of unsuccessful pore formation on a bare SiN membrane which ended in the massive accumulation of carbon at the point of e-beam exposure (after 10 min). (**F**) TEM image of successful pore formation using the SiO_2_ coated SiN aperture. Inset: TEM image of the SiO_2_ coating prior to graphene transfer and a schematic of the final substrate structure.

A compounding factor that acts alongside ad-atom migration is the topology of the graphene surface and the substrate material that the graphene lays on. Both 2D diffusional motion and the e-beam induced motion of ad-atoms can be hindered by surface roughness. Due to the holes found in holey carbon films, it is expected that the graphene will be most uneven on this substrate material. After the removal of the PMMA support material used during graphene transfer, it is expected that the graphene will relax onto the substrate with an uneven topology. Under TEM observation it was typically found that the focal plane of the free-standing graphene was different than the carbon-supported graphene (Figure 5A). Therefore these topology barriers would exist across the entire holey carbon film and limit the diffusion of ad-atoms. Uneven topology would therefore promote the formation of graphene nanopores since the rate of knockout would be greater than the rate of ad-atom self-healing. This is in general agreement with the fact that holey carbon films provided the best results in terms of the success rate of nanopore formation. SiN on the other hand is arguably the smoothest substrate with a surface roughness of 0.3 nm [53] and only a single defect which is the aperture that supports the graphene. SiN substrates also required the largest apertures in order for nanopore fabrication to be successful. The topology and temperature dependent migration of carbon ad-atoms (Figure 5D) seems to most accurately model that accounts for the differences in graphene nanopore formation.

Since holey carbon films cannot be used for biosensing applications, there was a desire to develop a surface modification to SiN that could reduce the aperture size needed for successful nanopore formation. The smallest aperture that resulted in pore formation was 624 nm whereas 533 nm resulted in no pore (Figure 5E). The first choice was to use a silicon dioxide coating which can be deposited either by a gas-phase deposition process or wet chemical growth process. The wet chemical sol-gel method was chosen since it is more likely that the solution phase reaction will produce a more uneven surface topology. The resulting substrate had ~70 nm of SiO_2_ deposited on the SiN surfaces as well as the FIB-drilled aperture. The final diameter, after SiO_2_ deposition, was 360 nm. After graphene transfer and loading into a TEM, a nanopore was formed after 100 s of e-beam drilling. The ability to reduce the overall aperture size using SiO_2_ coatings therefore can be used to minimize the probability of having graphene defects (tears, holes, contamination) on the free-standing portion of the graphene membrane. Additionally, the SiO_2_ coating can also improve noise characteristics of the device as shown previously [54]. It should also be noted that additional noise reductions are possible if silicon is replaced by quartz as the substrate material [55].

In terms of surface roughness, the SiN membrane coated with carbon via sputtering is expected to be smoother than the sol-gel grown SiO_2_. Typical root mean square (RMS) values for surface roughness of amorphous carbon-coated surfaces are ~0.6 nm. This is only slightly higher than the surface roughness of bare SiN deposited by low pressure chemical vapor deposition which has RMS values of approximately 0.3 nm [53]. Any increase in surface roughness when the SiN aperture was coated with carbon was likely offset by the increase in volume near the graphene which have competing effects on ad-atom migration. It should also be pointed out that the holey carbon film has an unknown surface roughness but the uneven topology instead comes from the fact that there are multiple holes (i.e., apertures) that contribute to the un-evenness of the graphene. The protocol used for SiO_2_ deposition from Wang et al. [44] reported a surface roughness RMS value of 1.61 nm which is the highest of all the SiN coatings used in this study. At this stage, it is unclear how the support material’s thermal conductivity, surface charge, and surface energy influence the movement of ad-atoms on the surface of graphene. It is also expected that Van der Waals forces between the substrate and the graphene would influence graphene’s material properties [56]. The support materials used here were silicon-based and the thin film properties do not vary as dramatically as needed to draw further conclusions. We believe the dominant factors to hindering ad-atom migration and therefore promoting nanopore formation include: (1) reducing the e-beam capture radius for ad-atoms and (2) increasing the surface roughness of the support material. 

**Figure 6:**
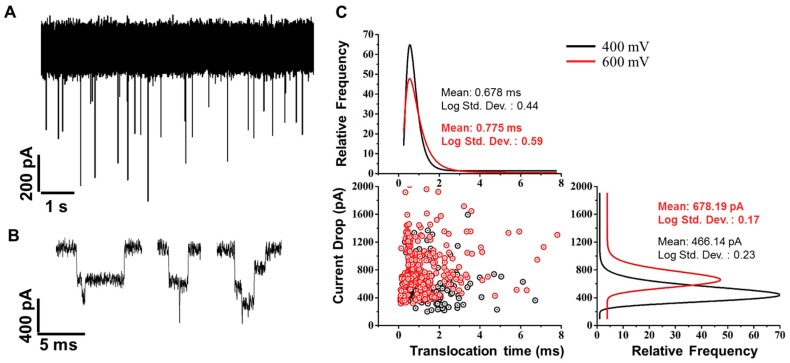
(**A**) Ionic current time trace after adding λ-DNA to the cis chamber of the flow cell. (**B**) Individual translocation events showing multiple current levels corresponding to the DNA molecules configuration. Both (**A**) and (**B**) were recorded at 400 mV voltage bias and with a 10 nm graphene pore at 1 M KCl. (**C**) Translocation event statistics of the same 10 nm graphene pore at 400 and 600 mV voltage bias. The number of events recorded was 298 and 422 for 400 and 600 mV, respectively.

Graphene nanopores, and more generally nanopores made from 2D materials, are powerful sensors of single molecules due to their atomically thin nature. With thin membranes, the ionic current blockade that is measured represents a smaller region of the molecule (i.e., the part of the molecule inside the pore). To demonstrate the sensing capabilities of these devices, λ-DNA was added to the cis chamber of the flow cell while ionic current was measured through a graphene nanopore with a diameter of 10 nm ([KCl] = 1 M). Upon adding DNA, transient reductions in the current are produced when a single DNA molecule traverses the pore (Figure 6A). Since the pore diameter is larger than the diameter of DNA, multiple configurations of DNA can be measured and each one produces a unique ionic current signature (Figure 6B) [9]. Many events also show what is now understood to be DNA knots which are characterized by short spikes within an event [57]. For event detection, we used threshold based search algorithm which captures the maximum current drop values of the events. The extracted event values are plotted in the current drop-translocation time scatter plot in Figure 6C. We conducted DNA translocation experiments at transmembrane voltages 400 mV and 600 mV, which correspond to black and red colored events respectively in the scatterplot. The marginal histograms show the lognormal curve fit to the current drop and translocation time data for both the voltages. As expected, we observed higher current drop values for DNA translocations at 600 mV (mean: 678.19 pA) compared to 400 mV (mean: 466.14 pA). We did not see significant difference in translocation time at the two voltages, which can be attributed to the high variability in event duration due to DNA folding on itself (Figure 6B). This work demonstrates the practical utility of graphene nanopores and the benefit of characterizing the parameters of fabrication. Although the amount of carbon on the graphene surface will vary according to the transfer procedure used by a laboratory, this work outlines several parameters which can be systematically changed in order to make functional devices; namely aperture size, membrane material, and surface roughness. The devices used to record λ-DNA had a SiN aperture size of ~500 nm, and was subsequently coated with ~100 nm of SiO_2_. An alternative approach is to make larger SiN apertures (i.e., 900–1000 nm) which were also used in previous work to detect λ-DNA [57]. 

## 4. Conclusions 

In conclusion, e-beam sculpting of graphene is not just dependent on the ability to displace atoms but also the ability to hinder the self-healing properties of graphene. Using relatively lower e-beam fluxes from a thermionic electron source, the C-atom knockout rate seems to be comparable to the rate of C-atom ad-atom attraction and accumulation at the e-beam/graphene interface (i.e., R_knockout_ ≈ R_EBID_). Working at this unique regime has allowed the study of C-atom ad-atom migration as well as the influence of various substrate materials on graphene pore formation. Since the e-beam is able to elevate the temperature of a material, in combination with the fact that graphene has a high thermal conductivity, there seems to be long-range effects on accumulation of surface ad-atoms. The temperature rise in the vicinity of the e-beam is likely to play a role in the diffusion of surface ad-toms. In the model that was presented in this work, diffusion must be overcome in order to be attracted towards the e-beam. Therefore, higher temperatures promotes diffusion and limits the ability accumulate ad-atoms near the e-beam. Since the drawing and storing of heat depends both on conductivity and the amount of supporting material, thin carbon grids led to successful drilling at virtually any aperture size whereas SiN membranes requires aperture sizes >600 nm. The fabrication of graphene nanopores, and more generally graphene nanostructures, has opened the door to a range of sensing applications and the development of novel devices over the years. This particular report aims at making graphene fabrication more robust by understanding the e-beam sculpting process. The understanding of these processes has led to several publications in the field of nanopore sensing including the ability to sense proteins using graphene [43] and the detection of 20 nucleotide long single stranded DNA [27].
